# Seismicity Pattern Changes before the *M* = 4.8 Aeolian Archipelago (Italy) Earthquake of August 16, 2010

**DOI:** 10.1155/2014/531212

**Published:** 2014-01-08

**Authors:** Salvatore Gambino, Antonino Laudani, Salvatore Mangiagli

**Affiliations:** ^1^Istituto Nazionale di Geofisica e Vulcanologia, Sezione di Catania Osservatorio Etneo, Piazza Roma 2, 95123 Catania, Italy; ^2^Dipartimento di Ingegneria, Università di Roma Tre, Via V. Volterra 62, 00146 Roma, Italy

## Abstract

We investigated the seismicity patterns associated with an *M* = 4.8 earthquake recorded in the Aeolian Archipelago on 16, August, 2010, by means of the region-time-length (RTL) algorithm. This earthquake triggered landslides at Lipari; a rock fall on the flanks of the Vulcano, Lipari, and Salina islands, and some damages to the village of Lipari. The RTL algorithm is widely used for investigating precursory seismicity changes before large and moderate earthquakes. We examined both the spatial and temporal characteristics of seismicity changes in the Aeolian Archipelago region before the *M* = 4.8 earthquake. The results obtained reveal 6-7 months of seismic quiescence which started about 15 months before the earthquake. The spatial distribution shows an extensive area characterized by seismic quiescence that suggests a relationship between quiescence and the Aeolian Archipelago regional tectonics.

## 1. Introduction

The quiescence of seismic activity has been defined as the notable decrease in the seismic activity against the average background. Temporal seismic observations have shown trends of seismic quiescence preceding large and moderate events [1, 2]. Successively Sobolev and Tyupkin [3, 4] proposed the region-time-length algorithm (RTL algorithm), a statistical method for the investigation of the seismic activity level preceding large earthquakes.

This method may evidence a decrease (quiescence) or an increase (activation) in the seismic activity against the average background [5]. The RTL method has previously been applied to earthquakes in Kamchatka and Caucasus [4, 6, 7], Japan (e.g., [7–9]), China [10, 11], Greece [12], Turkey [13], Taiwan [14], and India [15]. Some moderate Italian earthquakes have been studied by Di Giovanbattista and Tyupkin [16–18], Gentili and Bressan [19], and Gentili [20] by using this technique.

**Figure 1:**
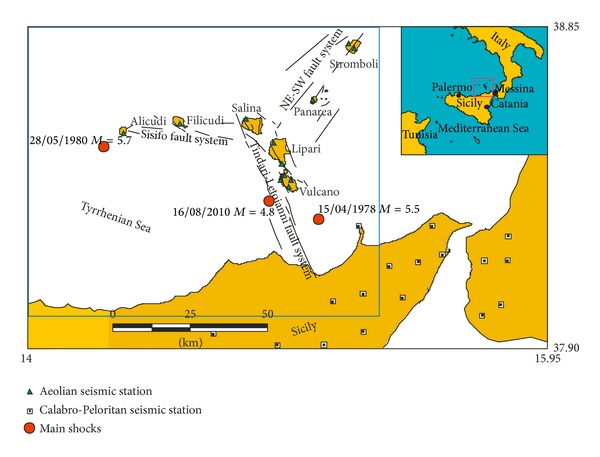
Map of the investigated area with main structural features and seismic network. The largest earthquakes, which occurred in Aeolian Archipelago in the last 50 years, are reported.

The Aeolian Archipelago (Figure 1) is located in the Southern Tyrrhenian Sea (Italy) and represents the manifestation of a submarine volcanic arc originating in the central sectors of the Tyrrhenian Sea during the Pliocene and successively migrating towards the southeast.

It can be subdivided into three sectors with a different structural and tectonic evolution [21]. In the western sector, comprising the Alicudi and Filicudi islands, the volcanic activity started at about 1.3 Ma [22] and ended at about 30–40 kyr. At present, the seismicity occurs in the crust along the WNW-ESE Sisifo fault system (Figure 1). The eastern sector, which comprises Panarea and Stromboli islands and where volcanism developed from 0.8 Ma ago and is still active and is affected by a prevailing NE-SW striking fault system. The central sector includes the islands of Salina, Lipari, and Vulcano. Here, the volcanism began at 0.4 Myr [23] and is still active (last eruption 1888–1890) at Lipari and Vulcano (e.g., [24–27]).

These volcanoes are aligned along a lithospheric NNW-SSE fault system, the Aeolian-Tindari-Letojanni fault system (Figure 1) with right-lateral to oblique kinematics along which the seismicity is roughly aligned (e.g., [28–30]). Earthquakes occur mostly in the upper 20 km of the crust [31]; in particular, the seismicity west of Tindari-Letoianni fault system is distributed in a 7–18 km interval of depth, whereas earthquakes of Lipari-Vulcano eastern area are not deeper than 12-13 km [32].

The southern Tyrrhenian area is characterized by seismicity with maximum magnitude in the range of 5-6; in the last 50 years two strong events have been recorded: *M* = 5.5 (15/04/1978) and *M* = 5.7 (28/05/1980) (Figure 1, [33]). These two moderate earthquakes have marked an increase of the regional dynamics that, according to Chiodini et al., 1992 [34], and Montalto, 1996 [35], caused the reactivation of the volcanic system on Vulcano. Moreover the occurrence of an earthquake of regional significance shortly before the last eruption of Vulcano [36] confirms that a moderate seismic event could initiate a rapid magma ascent.

The seismicity recorded from 1999 to 2011 comprises events with *M* < 5.0 and the 16, August, 2010 (*M* = 4.8), one represents the event with the highest magnitude recorded. In this study, the region-time-length (RTL) algorithm has been implemented to the catalogue of earthquakes which occurred in the period from 2000 to 2010 and we discuss the phases of seismic activation and quiescence preceding the *M* = 4.8 event in 2010.

## 2. Data

Since the late ‘70s, continuous seismic monitoring activity in the Aeolian Archipelago has been performed by a permanent seismic network made up of a few analogical 3C stations. Starting from the ‘80s, the network was augmented with other stations deployed over the entire Aeolian Archipelago and equipped with short-period seismometers, having a natural frequency of 1 Hz. During 2005 and 2007, almost all the stations were replaced by new digital 24-bit ones, equipped with broadband (40 s) three-component sensors, with a dynamic range of 144 dB. To date, the Aeolian permanent seismic network, managed by INGV-CT (Istituto Nazionale di Geofisica e Vulcanologia-Sezione di Catania), consists of 12 three-component digital seismic stations (Figure 1). In order to reduce the azimuthal gap, events location is obtained also using the stations deployed in the Calabro-Peloritan area and on the northern flanks of Mt. Etna (Figure 1). Furthermore, where possible, we added data from the INGV national permanent seismic network.

**Figure 2:**
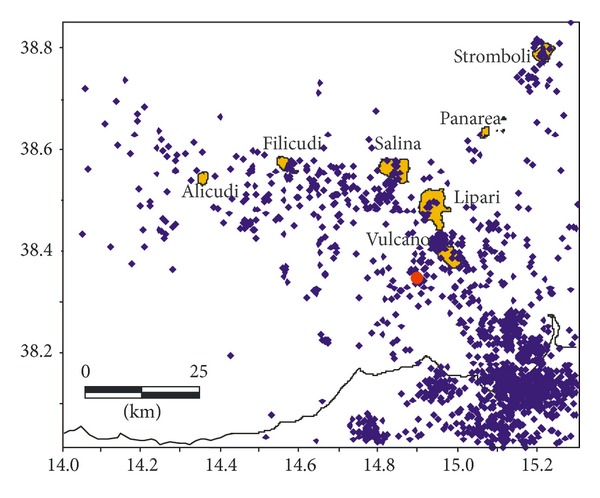
Seismic activity recorded in the Aeolian Archipelago area during the August 1999–December 2011 period. The dataset comprises 1680 earthquakes recorded from August 1999 to 2011; the red circle shows location of the 16, August, 2010, *M* = 4.8 earthquake.

We considered an area of 100 × 80 km with a latitude between 38.00 and 38.85 and a longitude of 14.00 and 15.30. The dataset used in this study comprises 1680 crustal earthquakes recorded from August 1999 to 2011 with magnitude 1.0 ≤ Md ≤ 4.8, whose location, performed for surveillance purposes, is obtained by using the Hypoellipse code [37] (Figure 2). The mean errors of the analytical locations are, respectively, 0.95 km for the epicentral coordinates and 1.15 km for focal depth; the mean root mean square (RMS) is 0.16 s.

The main event of the catalogue occurred at 12.54 GMT of the 16, August, 2010, when Aeolian Archipelago was shaken by an earthquake of an estimated 4.8 magnitude. The hypocenter of the earthquake was situated 8 km west-south-west of the island of Vulcano at a depth of 13.0 km b.s.l. (Figure 1). The earthquake was felt on the northern coast of Sicily and in the cities of Palermo, Catania, and Messina. The earthquake triggered some landslides at Lipari, a rock fall on the flanks of Vulcano Lipari and Salina. In the village of Lipari minor damages to buildings and roads were reported and some beaches were closed for safety reasons.

## 3. Method

The analysis of the earthquake dataset has been performed by using the well-established method known as RTL algorithm [4, 8] which uses three parameters, namely, *R* (region around the earthquake epicenter), *T* (time), and *L* (rupture length). The fundamental idea of RTL algorithm is to assign a weighting RTL value to a given spatiotemporal value (*x*, *y*, *z*, *t*), which comes from events occurring in a prescribed space-time window within the characteristic distance and time. An RTL parameter is defined as the product of *R*, *T*, and *L* describing the influence weights of location, occurrence time, and magnitude as

(1) R(x,y,z,t)=[∑i:1nexp(−riro)I(ri≤2ro)I(t−ti≤2to)   ×I(di≤do)I(Mi≥Mmin)] −Rbk(x,y,z,t),T(x,y,z,t)=[∑i:1nexp(−t−tito)I(ri≤2ro)I(t−ti≤2to)   ×I(di≤do)I(Mi≥Mmin)] −Tbk(x,y,z,t),L(x,y,z,t)=[∑i:1n(−liri)I(ri≤2ro)I(t−ti≤2to)   ×I(di≤do)I(Mi≥Mmin)] −Lbk(x,y,z,t),

where *l*_*i*_ is the rupture dimension (a function of magnitude *M*_*i*_); *t*_*i*_ is the occurrence time of the *i*th earthquake; *r*_*i*_ is the distance from the position (*x*, *y*, *z*) to the epicenter of the *i*th event; *r*_0_ and *t*_0_ are the characteristic distance and time associated with the spatiotemporal criteria; *d*_*o*_ is the cut-off depth; and *n* is the number of events satisfying the following criterion:

*M*_*i*_ ≥ *M*_min_ (*M*_*i*_ is the magnitude of *i*th event and *M*_min_ is the cut-off magnitude ensuring the completeness of the earthquake catalogue); *r*_*i*_ ≤ *R*_max_ = 2*r*_*o*_; and *t* − *t*_*i*_ = *T*_max_ = 2*t*_*o*_.

For the rupture dimension *l*_*i*_ the following expression is used [16]:

(2) $$\begin{array}{c} l_{i}=\exp\left(0.44*M_{i}-1.289\right). \end{array}$$

**Figure 3:**
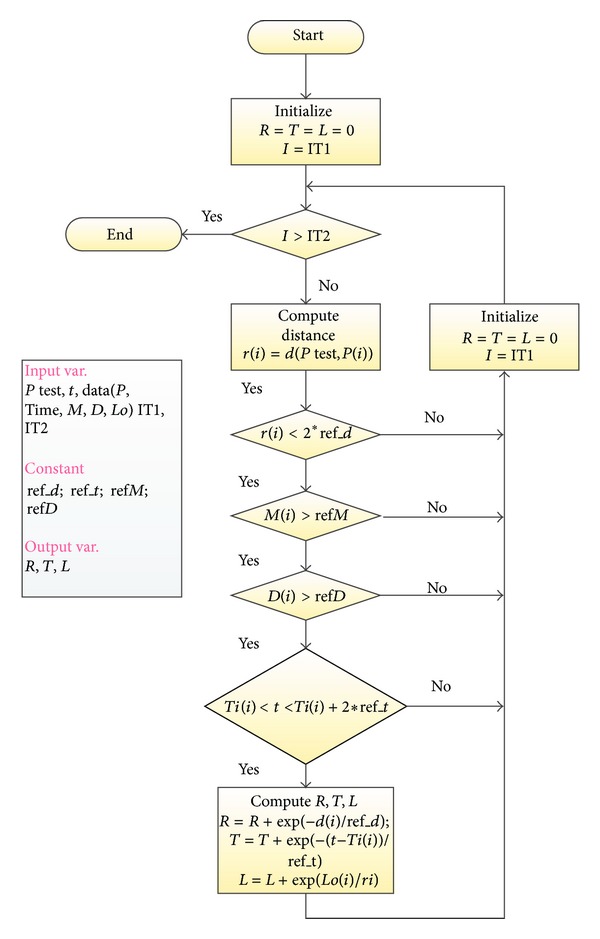
Flowchart that documents the algorithm used on Matlab for RTL analyses. The input variable “data” contains all the information about the events to be analyzed. *P* test and *t*, are respectively, the position and the time to be studied, whereas IT1 and IT2 individuate the interval of time to be considered for the RTL analysis. Clearly the constants “ref_*d*,” “ref_*t*,” “ref*M*,” and “ref*D*” correspond, respectively, to *r*_*o*_, *t*_*o*_, *M*_min_, and *d*_*o*_.

*R*_bk_, *T*_bk_, and *L*_bk_ are the background values of *R*, *T*, and *L*, respectively, obtained as the expected values in the time interval considered for the analyzed position. RTL parameter describes the deviation from the background level of seismicity and is expressed in units of the standard deviation. A negative RTL value indicates a lower seismicity and a positive RTL value indicates a higher seismicity compared to the background. Clearly, both a temporal and a spatial analysis of RTL can be performed and some authors often use the spatial average value for the RTL parameters calling it *Q* parameter [11]. The algorithm for the computation of *R*, *T*, *L*, and *Q* parameters has been implemented in the MATLAB environment (Figure 3), allowing simple management and plotting of the results.

## 4. RTL's Calculation and Results

The RTL analysis needs to be applied to declustered catalogues, where aftershocks are removed [9]. In order to decluster the INGV catalogue, we applied the Reasemberg [38] algorithm implemented in Zmap software [39].

The Reasenberg algorithm defines a seismic sequence as a chain of events linked to each other by spatial and temporal windows. The variables are *r*_fact_, the factor for the interaction radius of dependent event, *τ*_min_, the look-ahead time for un-clustered events in days, *τ*_max_, the maximum look-ahead time for clustered events in days, and *P*, a measure of the confidence that the next event in the sequence is being observed.

For declustering we used the default parameters (*r*_fact_ = 10, *τ*_min_ = 1, *τ*_max_ = 10, and *P* = 0.95) obtaining a catalogue of 1324 events.

Moreover the magnitude of completeness has been evaluated for the catalogue by using the Gutenberg-Richter relation of earthquake frequency and magnitude.

The completeness of the data entries depends on the characteristics of the seismic network. The geometry, sensitivity, and resolution of the seismic network quantify different parts of the region in order to judge the behavior of the seismic regimes based on the representation of minimum magnitude.

We applied the Gutenberg-Richter relation to search the *M*_min_, representative magnitude for the present earthquake time series of the Aeolian Archipelago region.

**Figure 4:**
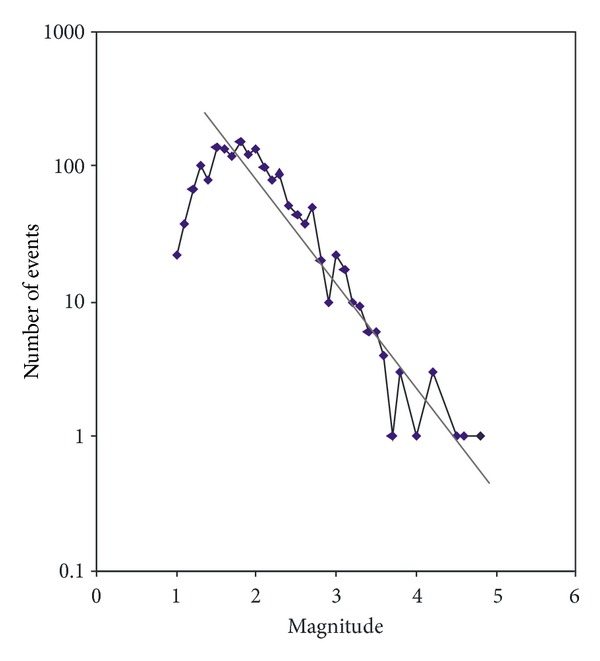
*B*-value estimation of the complete earthquake dataset (August 1999 to 2011) after removing the aftershocks in the Aeolian Archipelago region.

The power law of Gutenberg-Richter fits the earthquake energy distribution as a linear plot of recurrence. The bending of the linear plot for the smaller magnitude earthquake gives an indication of incompleteness of the catalogue below a specified magnitude. This specified magnitude is the minimum or the threshold magnitude for the studied area.  Figure 4 shows the earthquake frequency magnitude plot. It may be noted from here that the data are complete for earthquakes of *M* = 1.8. After removing events with *M* < 1.8 the remaining data comprised 838 events which were used for the present study to estimate the RTL variation.

We calculated the RTL and *Q* parameters [9, 13], that is, the possible time and spatial variation of the seismic quiescence, and to this end we made some choices about the input parameters; if we consider, for example, the *M* = 7.3 earthquake, which occurred in the western region of Tottori prefecture, Japan, on 6, October, 2000, Huang [9] adopted a distance *r*_0_ = 50 km, *t*_0_ = 1 year, and a focal depth (*d*_0_) of 30 km.

Shashidhar et al. [15], for moderate earthquakes (*M* = 5.0) in a small area (20 km × 30 km), tried different values for parameters adopting the following values: *r*_0_ = 10 km, *t*_0_ = 25 days, and *d*_0_ = 20 km.

In order to obtain the RTL, we have considered location of the 16, August, earthquake (*M* = 4.8) and the 1, January, 2008–15, August, 2010 (958 days), period.

**Figure 5:**
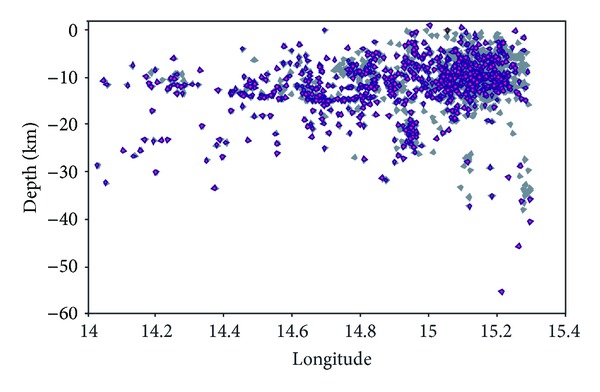
Cross-section of the selected seismicity catalogue to show depth distribution of all seismicity (grey diamonds) and after removing aftershocks and *M* < 1.8 (purple diamonds).

**Figure 6:**
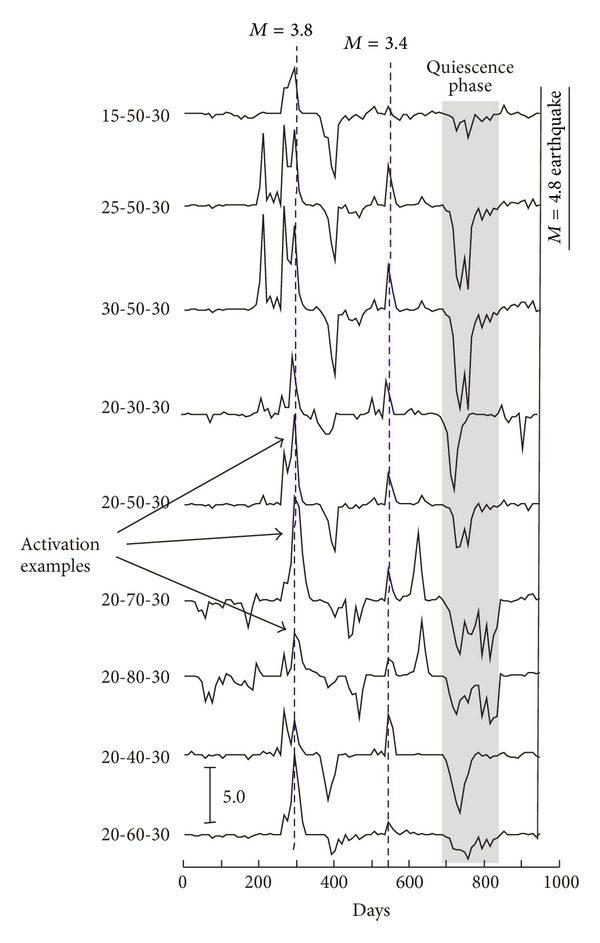
Temporal variation of the RTL at the epicenter of the 16, August, 2010, *M* = 4.8 earthquake. The variation in RTL anomaly is obtained at this location with different values of parameters reported on the left of the graphs. The three values are referred to as *r*_*o*_, *t*_*o*_, and *d*_*o*_. Starting point is 1, January, 2008 and duration is 958 days. Some examples of seismic activation are indicated by arrows.

**Figure 7:**
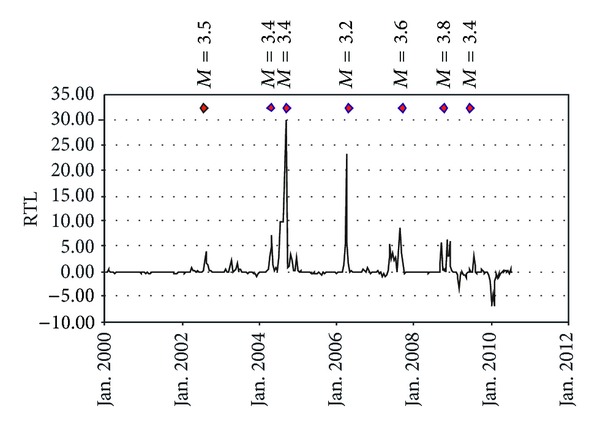
2000–August 2010 time variation of the RTL at the epicenter of the 16, August, 2010, *M* = 4.8 earthquake by using *r*_*o*_ = 25, *t*_*o*_ = 50, and *d*_*o*_ = 30 as input parameters. Red diamonds identify the occurrence times of the 3.2 ≤ *M* ≤ 3.8 earthquakes located nearby (10–15 km) the 16, August, 2010, epicenter.

We set a focal depth of 30 km considering that almost all earthquakes (98.7%) are not more than 30 km deep (Figure 5) and we tried different values of *r*_0_ and *t*_0_ (Figure 6). RTL algorithm does not show large differences between the different curves (Figure 6); we adopted the following model parameters: a characteristic distance *r*_0_ = 25 km and *t*_0_ = 50 days. Finally, we also ran (Figure 7) the RTL algorithm for the entire 2000–August 2010 period.

## 5. Discussion

In many parts of the world, the RTL method has been used for larger regions and longer seismic catalogues, obtaining valid observations on the quiescence phenomenon prior to large earthquakes [11, 40]. In this study, the RTL algorithm has been implemented in the MATLAB environment and tested to a triggered earthquake time series occurring in the Aeolian Archipelago (Italy), a relatively small area (100 km × 80 km) characterized by a moderate seismicity. A phase of seismic quiescence (between the 700th and the 850th day in Figure 6) was detected by the *Q* parameter around the epicenter of the 16, August, 2010, earthquake. The seismic quiescence spans the June–December 2009 period, ending 8-9 months before the *M* = 4.8 earthquake.

Figures 6 and 7 show the presence of short-time positive changes (activations) during the 2000–2010 period. These short changes are linked to the occurrence of 3.2 ≤ *M* ≤ 3.8 earthquakes located nearby (10–15 km) the *M* = 4.8 epicenter. Moreover some modest and rapid negative changes are also visible.

All these short-time variations may be related to an imperfect removing of the aftershocks in the catalogue and/or to the adopted parameters.

Six-seven-month quiescence ending 8-9 months before the earthquake is in accordance with duration of the quiescence (0.6 to 3 years) and time shift from the end of the quiescence to the earthquake (0 to 2.9 years) was found by Gentili [20] for several Italian sectors.

**Figure 8:**
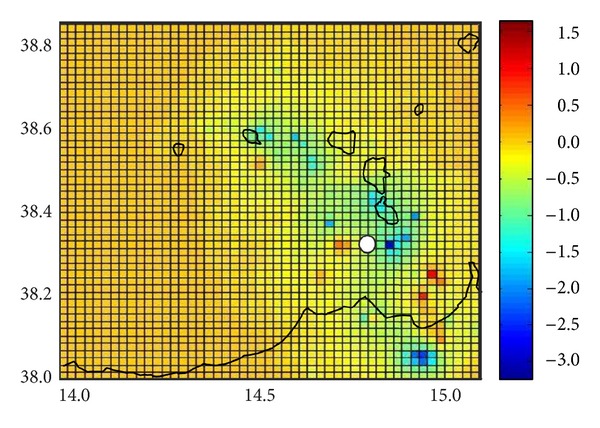
Spatial variation of RTL in the Aeolian Archipelago area during the observed quiescence period (June–December 2009). The scale on the right corresponds to the RTL value in the units of the standard deviation. The white circle shows location of the 16 August 2010, *M* = 4.8 earthquake.

Finally, in order to investigate its possible spatial variation, we calculated the *Q* parameter in the quiescence period (June–December 2009) for the entire area (Figure 8). To this end, the territory has been divided into 2,500 cells, each one with an area of ca. 4 km^2^.

The main area covered by the quiescence (ca. 200 km^2^) comprises a sector around Vulcano island. The *M* = 4.8 earthquake occurred in this area, which is about 4 km west of the pixel with lower (−3.3) *Q* value. However, an area between Salina and Filicudi of ca. 80 km^2^ and a small sector (ca. 40 km^2^) in the north of Sicily also show negative *Q* values. Areas covered by the quiescence agree with sectors affected by Sisifo and Tindari-Letojanni fault system.

Considering the *M* = 4.8 location (Figures 1 and 8) we would expect an quiescence focussed only on Tindari-Letojanni fault system. As different tectonic zones have different background seismicity [13] our results suggest a continuity between the two structures and a relationship between the quiescence recorded before the *M* = 4.8 and overall Aeolian Archipelago regional tectonics.

## 6. Conclusions

The results obtained reveal a seismic quiescence phase before an *M* = 4.8 earthquake with an extensive (ca. 320 km^2^) spatial distribution which comprises the triggered zone. However the area covered by the quiescence seems large in order to affirm that the method presented here is an effective tool of improving the significance and reliability of earthquake precursors.

These features are consistent with the results obtained by different authors by using RTL worldwide and encourage us to improve RTL analyses on other earthquakes by testing different parameters in order to evaluate the future possibility of moderate earthquake occurrence in this region that could also have an impact on the volcanic system of Vulcano.

## References

[1] Wyss M, Habermann RE (1988). Precursory seismic quiescence. *Pure and Applied Geophysics*.

[2] Wiemer S, Wyss M (1994). Seismic quiescence before the Landers (M = 7.5) and Big Bear (M = 6.5) 1992 earthquakes. *Bulletin of the Seismological Society of America*.

[3] Sobolev GA, Tyupkin YS (1996). New method of intermediate-term earthquake prediction. *Seismology in Europe*.

[4] Sobolev GA, Tyupkin YS (1997). Low-seismicity precursors of large earthquakes in Kamchatka. *Volcanology and Seismology*.

[5] Wu YM, Chen CC, Zhao L, Chang CH (2008). Seismicity characteristics before the 2003 Chengkung, Taiwan, earthquake. *Tectonophysics*.

[6] Sobolev GA, Tyupkin YS (1999). Precursory phases, seismicity precursors, and earthquake prediction in Kamchatka. *Volcanology and Seismology*.

[7] Huang Q (2004). Seismicity pattern changes prior to large earthquakes—an approach of the RTL algorithm. *Terrestrial, Atmospheric and Oceanic Sciences*.

[8] Huang Q, Sobolev GA, Nagao T (2001). Characteristics of the seismic quiescence and activation patterns before the M = 7.2 Kobe earthquake, January 17, 1995. *Tectonophysics*.

[9] Huang Q (2006). Search for reliable precursors: a case study of the seismic quiescence of the 2000 Western Tottori prefecture earthquake. *Journal of Geophysical Research B*.

[10] Rong D, Li Y (2007). Estimation of characteristic parameters in region-time-length algorithm and its application. *Acta Seismologica Sinica*.

[11] Huang Q (2008). Seismicity changes prior to the Ms 8.0 Wenchuan earthquake in Sichuan, China. *Geophysical Research Letters*.

[12] Sobolev GA, Tyupkin YS, Zavialov A Map of expectation earthquakes algorithm and RTL prognostic parameter: joint application.

[13] Huang Q, Öncel AO, Sobolev GA (2002). Precursory seismicity changes associated with the Mw = 7.4 1999 August 17 Izmit (Turkey) earthquake. *Geophysical Journal International*.

[14] Chen CC, Wu YX (2006). An improved region-time-length algorithm applied to the 1999 Chi-Chi, Taiwan earthquake. *Geophysical Journal International*.

[15] Shashidhar D, Kumar N, Mallika K, Gupta H (2010). Characteristics of seismicity patterns prior to the M*∼*5 earthquakes in the Koyna Region, Western India—application of the RTL algorithm. *Episodes*.

[16] Di Giovambattista R, Tyupkin Y (1999). The fine structure of the dynamics of seismicity before M ≥ 4.5 earthquakes in the area of Reggio Emilia (Northern Italy). *Annali di Geofisica*.

[17] Di Giovambattista R, Tyupkin YS (2000). Saptial and temporal distribution of seismicity before the Umbria-Marche September 26,1997 eartquakes. *Journal of Seismology*.

[18] Di Giovambattista R, Tyupkin YS (2004). Seismicity patterns before the M = 5.8 2002, Palermo (Italy) earthquake: seismic quiescence and accelerating seismicity. *Tectonophysics*.

[19] Gentili S, Bressan G (2007). Seismicity patterns before MD C 4.1 earthquakes in the Friuli-Venezia Giulia (Northeastern Italy) and Western Slovenia areas. *Bollettino di Geofisica Teorica e Applicata*.

[20] Gentili S (2010). Distribution of Seismicity Before the Larger Earthquakes in Italy in the Time Interval 1994–2004. *Pure and Applied Geophysics*.

[21] de Astis G, Ventura G, Vilardo G (2003). Geodynamic significance of the Aeolian volcanism (Southern Tyrrhenian Sea, Italy) in light of structural, seismological and geochemical data. *Tectonics*.

[22] Gillot PY (1987). Histoire volcanique des Iles Eoliennes: arc insulaire or complexe orogenique anulaire?. *Documents et Travaux Institute Géologique Albert-de-Lapparent*.

[23] Beccaluva L, Gabbianelli G, Lucchini F, Rossi PL, Savelli C (1985). Petrology and K Ar ages of volcanics dredged from the Eolian seamounts: implications for geodynamic evolution of the Southern Tyrrhenian basin. *Earth and Planetary Science Letters*.

[24] Peccerillo A, Frezzotti ML, De Astis G, Ventura G (2006). Modeling the magma plumbing system of Vulcano (Aeolian Islands, Italy) by integrated fluid-inclusion geobarometry, petrology, and geophysics. *Geology*.

[25] Gambino S, Campisi O, Falzone G (2007). Tilt measurements at Vulcano Island. *Annals of Geophysics*.

[26] Gambino S, Guglielmino F (2008). Ground deformation induced by geothermal processes: a model for La Fossa Crater (Vulcano Island, Italy). *Journal of Geophysical Research B*.

[27] Alparone S, Cannata A, Gambino S, Gresta S, Milluzzo V, Montalto P (2010). Time-space variation of volcano-seismic events at La Fossa (Vulcano, Aeolian Islands, Italy): new insights into seismic sources in a hydrothermal system. *Bulletin of Volcanology*.

[28] Neri G, Barberi G, Orecchio B, Mostaccio A (2003). Seismic strain and seismogenic stress regimes in the crust of the Southern Tyrrhenian region. *Earth and Planetary Science Letters*.

[29] Neri G, Barberi G, Oliva G, Orecchio B (2005). Spatial variations of seismogenic stress orientations in Sicily, South Italy. *Physics of the Earth and Planetary Interiors*.

[30] Billi A, Barberi G, Faccenna C, Neri G, Pepe F, Sulli A (2006). Tectonics and seismicity of the Tindari fault system, Southern Italy: crustal deformations at the transition between ongoing contractional and extensional domains located above the edge of a subducting slab. *Tectonics*.

[31] Cannata A, Diliberto IS, Alparone S (2012). Multiparametric approach in investigating hydrothermal systems: the case of study of Vulcano (Aeolian Islands, Italy). *Pure and Applied Geophysics*.

[32] Gambino S, Milluzzo V, Scaltrito A, Scarfì L (2012). Relocation and focal mechanisms of earthquakes in the south-central sector of the Aeolian Archipelago: new structural and volcanological insights. *Tectonophysics*.

[33] Neri G, Caccamo D, Cocina O, Montalto A (1996). Geodynamic implications of earthquake data in the Southern Tyrrhenian sea. *Tectonophysics*.

[34] Chiodini G, Cioni R, Falsaperla S, Montalto A, Guidi M, Marini L (1992). Geochemical and seismological investigations at Vulcano (Aeolian Islands) during 1978–1989. *Journal of Geophysical Research*.

[35] Montalto A (1996). Signs of potential renewal of eruptive activity at La Fossa (Vulcano, Aeolian Islands). *Bulletin of Volcanology*.

[36] Mercalli G, Silvestri O (1891). L’eruzione dell’Isola di Vulcano incominciata il 3 agosto 1888 e terminata il 22 marzo 1890. *Annali dell’ufficio Centrale di Meteorologia e Geodinamica*.

[37] Gruppo Analisi Dati Sismici Catalogo dei terremoti della Sicilia Orientale—Calabria Meridionale (1999–2012). http://www.ct.ingv.it/ufs/analisti/catalogolist.php.

[38] Reasemberg PA (1985). Second-order moment of Central California seismicity, 1969–1982. *Journal of Geophysical Research*.

[39] Wiemer S (2001). A software package to analyze seismicity: ZMAP. *Seismological Research Letters*.

[40] Sobolev GA (2004). Microseismic variations prior to a strong earthquake. *Izvestiya, Physics of the Solid Earth*.

